# Analysis of Typical Inclusion Evolution and Formation Mechanism in the Smelting Process of W350 Non-Oriented Silicon Steel

**DOI:** 10.3390/ma18061188

**Published:** 2025-03-07

**Authors:** Jiagui Shi, Libin Yang, Bowen Peng, Guoqiang Wei, Yibo Yuan

**Affiliations:** Metallurgical Technology Institute, Central Iron and Steel Research Institute Co., Ltd., Beijing 100081, China; shijiag1626@163.com (J.S.);

**Keywords:** non-oriented silicon steel, typical inclusions, RH refining, evolution, thermodynamic analysis

## Abstract

The production of silicon steel involves complex metallurgical processes, where the kind, composition, size, and quantity of the inclusions generated affect the silicon steel properties. This article is based on the smelting process for W350 non-oriented silicon steel produced by a certain factory. By systematically sampling, at key nodes of the converter–RH refining–tundish smelting process, the change in cleanliness of molten steel in the whole smelting process, the evolution of typical inclusions, and the transformation rules for the precipitated phase were analyzed by means of SEM-EDS, ASPEX, and Thermal-Calc. The results indicate that the total oxygen mass fraction in the steel decreases by more than 95% after deoxidation alloying, and the average oxygen mass fraction in the RH outbound steel is 0.0012%. While the nitrogen mass fraction shows a rising trend as a whole, the average nitrogen mass fraction in the tundish steel reaches approximately 0.0014%. Before RH refining, large Al_2_O_3_–CaO–SiO_2_ and Al_2_O_3_–CaO–SiO_2_–MgO composite inclusions are the main inclusions. MnO and Al_2_O_3_–SiO_2_–MnO inclusions are the main inclusions after RH inlet and RH decarburization. After RH deoxidation with aluminum, the inclusions were almost entirely transformed into Al_2_O_3_ inclusions. After RH alloying, with the content of Si and Mn increased, the inclusions transformed into Al_2_O_3_–SiO_2_–MnO inclusions. The number of inclusions from RH desulfurization to the RH outbound stage declined significantly, and composite inclusions containing CaS and precipitates such as AlN and MnS began to appear. The inclusions’ main types were Al_2_O_3_–MgO–CaS, AlN–MnS, AlN, and Al_2_O_3_–MgO. The inclusions inside the tundish were the same, but the numbers were slightly increased due to the secondary oxidation of molten steel. More than 80% of the oxide inclusions in the whole process were between 1 μm and 5 μm in size. The average size and the number of inclusions per unit area reached 5.45 μm and 63.1 per mm^2^, respectively, after RH deoxidation, and respectively decreased to 3.71 μm and 1.9 per mm^2^ during the RH outbound stage, but both increased slightly in the tundish. Thermodynamic calculation shows that Al_2_O_3_–MgO inclusions are formed when *w*([Mg]) > 0.0033% in molten steel at 1873 K. Under the actual temperature of 1828K and *w*([Al]s) = 0.6515%, the range of *w*([Mg]) corresponding to the stable existence of Al_2_O_3_–MgO is between 0.0053% and 0.1676%. The liquidus temperature of W350 non-oriented silicon steel is 1489 °C. MnS and AlN inclusions are precipitated successively with the solidification of molten steel, and the precipitation temperatures are 1460.7 °C and 1422.2 °C, respectively. As the temperature decreases, the sequence of inclusion precipitation calculated in liquid was as follows: Al_2_O_3_–CaO → 2Al_2_O_3_–CaO + MnS → 6Al_2_O_3_–CaO → Al_2_O_3_ + AlN + MnS + CaS.

## 1. Introduction

In recent years, driven by the rapid rise of the new energy automobile industry and efforts to upgrade the energy efficiency of industrial motors, non-oriented silicon steel has become the core functional soft magnetic material in the process of electromagnetic energy conversion [1]. It plays an important role in electronic production, power construction, aerospace, and military product development due to its performance, high strength, high magnetic induction, and low iron loss [2,3,4]. The high mass fraction of silicon and aluminum in non-oriented silicon steel is conducive to reducing iron loss but has an adverse effect on the steel’s brittleness and processability; moreover, it promotes the formation of inclusions during the smelting process [5,6]. Inclusions in silicon steel have a significant effect on the magnetic properties of steel products [7,8]. Their formation is closely related to the raw materials used, as well as smelting, casting, rolling, and other processes [9]. In particular, deoxidation, alloying, desulfurization, argon blowing, and other smelting processes will directly affect the composition, quantity, morphology, and size of inclusions in steel [6,10].

Some authors have analyzed the evolution of inclusions in various steel smelting processes [11,12,13]. For silicon steel, it is more about the change of inclusions in a certain stage of smelting or the evolution of a specific inclusion in the smelting process. Hu et al. [14] found that the oxide types in non-oriented silicon steel W800 evolved from spherical or near-spherical SiO_2_ and SiO_2_–MnO, with a small amount of SiO_2_ bound to Al_2_O_3_ and MgO–Al_2_O_3_ after the addition of aluminum alloy. Cao et al. [15] sampled and analyzed the whole process for DG47A non-oriented silicon steel. They found that the oxide types from Ruhrstahl and Heraeus vacuum degassing (hereinafter referred to as RH) and after decarburization were mainly larger-size polygonal SiO_2_, which evolved into elongated Al_2_O_3_ and SiO_2_ inclusions after aluminum addition. After the alloying and vacuum end, these evolved into Al_2_O_3_–MgO and MnS-containing composite inclusions. There are no single-phase Al_2_O_3_ inclusions in the vacuum end. The tundish and casting billet are mainly Al_2_O_3_–MgO composite inclusions bound by spherical CaS, MnS, and AlN. Sun et al. [16] calculated and simulated the formation conditions for Al_2_O_3_–MgO inclusions and slag entrapment behavior in molten steel during the smelting process for non-oriented silicon steel. Li et al. [17] studied the change in inclusions in a non-oriented silicon steel slab under the conditions of different deoxidation processes (Si or Al deoxidation). According to the research, the density and the inclusion-occupied area in an Al deoxidation slab were greater than those in a Si deoxidation slab, but the stability of cleanliness was inferior to that on a Si deoxidation slab.

It can be seen that the evolution of inclusions in the smelting process for non-oriented silicon steel of different grades is completely different. In order to further improve the product quality and performance of W350 non-oriented silicon steel and expand its high-end market application, this paper systematically sampled different stages of its smelting process. The formation mechanism and evolution law of typical inclusions, including the types, sizes, morphology, and quantities, in the whole process for non-oriented silicon steel smelting were comprehensively investigated by sampling analysis and thermodynamic calculations. This paper also discusses the change in the molten steel cleanliness of the whole smelting process. In particular, the change in typical inclusions in each stage of the RH refining process is systematically analyzed, providing a scientific theoretical basis and reference for the further control and removal of inclusions in practical production.

## 2. Materials and Methods

### 2.1. Materials

The smelting process for W350 non-oriented silicon steel produced by the factory involves the following steps: “hot metal pretreatment → 300 t top-bottom combined blowing converter → argon blowing station → RH refining → continuous casting”. The hot metal from the blast furnace undergoes three slag removal operations and two stirring processes. Most notably, the molten steel is not deoxidized during the converter tapping process, which utilizes boiling tapping. The RH refining treatment process includes the following stages: “decarburization → deoxidation → alloying → desulfurization” with a deep vacuum maintained for over 12 min, followed by aluminum deoxidation for 3 to 5 min. The alloy is added according to batch, and the circulation time after alloy addition is no less than 3 min. The flow rate for the addition of RH desulfurizer is controlled at 100–120 kg/min, with a net cycle time after complete addition of no less than 5 min. Following the completion of the vacuum process and RH outbound, the molten steel is transported to the ladle turret and subsequently sent to the tundish for further treatment. The chemical composition control standards for W350 non-oriented silicon steel in the tundish are detailed in Table 1.

### 2.2. Sampling and Analysis Method

**Figure 1:**
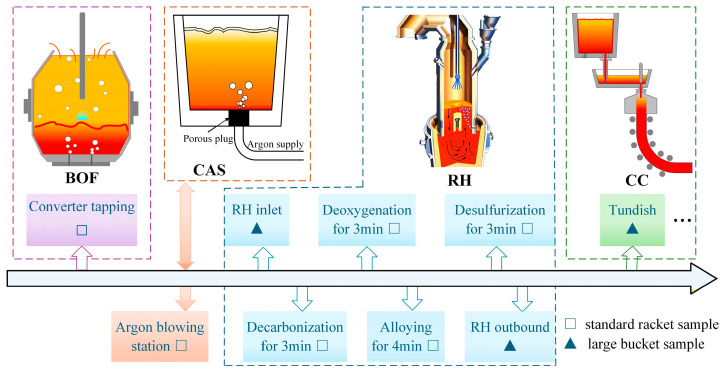
Sampling scheme for the whole smelting process of W350 non-oriented silicon steel.

To comprehensively investigate the evolution laws and formation mechanisms of typical inclusions in molten steel during the W350 non-oriented silicon steel smelting process, and to ensure the reliability of sampling and analysis results, four batches of molten steel samples were continuously taken during the same pouring planning. The samples were collected at the end-point of the converter, the argon blowing station, and the RH inlet, as well as after RH vacuum decarburization, aluminum deoxygenation, the first batch of alloying, desulfurization, RH outbound, and the tundish. Specifically, bucket samples were collected at the RH refining inlet, RH outbound, and the tundish to accurately assess their cleanliness. The specific sampling scheme is shown in Figure 1.

**Figure 2:**
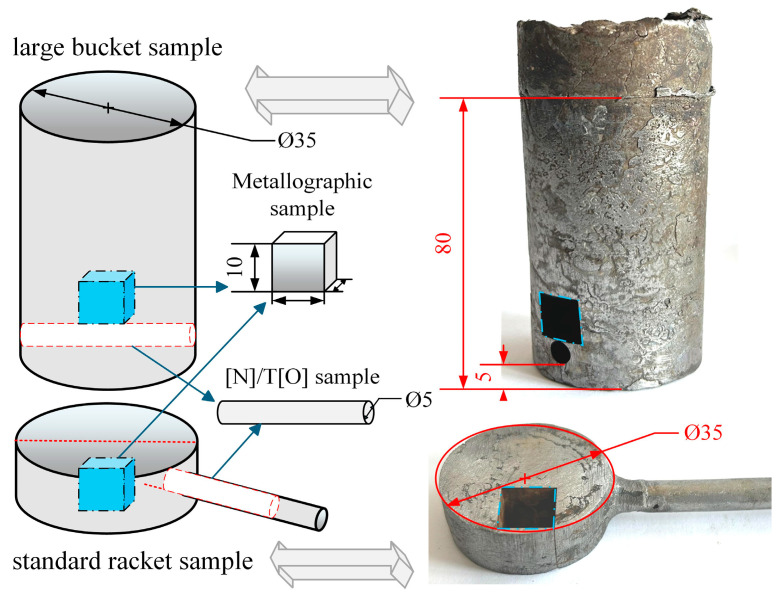
Schematic diagram of sample machining.

Systematic sampling was carried out according to the above scheme, where the large bucket sample (Φ35 mm × 80 mm) could extract more molten steel compared to the standard racket sample (Φ35 mm × 10 mm), as the bubbles in the molten steel could be fully floated during sampling. This resulted in more accurate test results. Some square metallographic samples with a specification of 10 mm × 10 mm × 10 mm were processed by wire cutting machine at the middle position of the standard racket sample and the middle position of the bucket sample above 5 mm from the bottom. Some nitrogen–oxygen analysis specimens, 5 mm in diameter, were cut near the bottom of the bucket sample and then sanded and polished for analysis. The specific processing diagram is shown in Figure 2.

The composition of molten steel in each process was analyzed using Inductively Coupled Plasma Atomic Emission Spectrometry (ICP-AES), which can achieve measurement temperatures ranging from 6000 K to 8000 K. The relative standard deviation of the quantitative analysis results is typically maintained within 1% to 5%. To ensure the accuracy of the test results, samples subjected to automated scanning analysis were subsequently reused for ICP analysis. The average composition of molten steel in the RH outbound and tundish is presented in Table 2, which is utilized to calculate the formation conditions of the typical inclusions in molten steel and the thermodynamic conditions of the precipitated phase during solidification. The composition, quantity, and size distribution of different inclusions in metallographic samples were analyzed using Automated Scanning Probe Exfoliation (ASPEX), which has a scanning accuracy for inclusions larger than 1 μm, and a scanning area of 20 mm^2^. The content of oxygen and nitrogen in molten steel from different processes was detected by the inert gas fusion–infrared absorption method. The morphology and type of inclusions in molten steel were analyzed using a Scanning Electron Microscope (SEM) and Energy Dispersive Spectrometer (EDS). The elemental analysis accuracy of EDS generally ranges from 1% to 5%, with a detection limit of 0.1%, making it suitable for semi-quantitative analysis. Finally, the precipitation mechanism of typical inclusions in molten steel was analyzed and calculated using the thermodynamic software Thermal-Calc 2022b, alongside an analysis of the potential sources of various inclusions.

## 3. Results

### 3.1. Changes in w(T[O]) and w([N]) in Molten Steel

The content of total oxygen (hereinafter referred to as T[O]) and nitrogen (hereinafter referred to as [N]) are usually used as crucial indices to evaluate the cleanliness of molten steel [18]. Specifically, the T[O] content in the molten steel comprises dissolved oxygen and oxygen content in non-metallic inclusions. The W350 non-oriented electrical steel contains a high content ratio of silicon (Si) and aluminum (Al). After alloying, the dissolved oxygen content in the molten steel exhibits minimal change and approaches zero. Thus, it can be considered that nearly all oxygen in the steel is present in the form of oxide inclusions. Consequently, variations in the mass fraction of T[O] in the steel can accurately reflect the cleanliness level of the molten steel [14]. Additionally, the [N] in molten steel typically originates from air; therefore, the change value of [N] in molten steel from the ladle to the tundish can be utilized to assess the degree of secondary oxidation in the molten steel and to analyze potential issues in protective casting [19].

**Figure 3:**
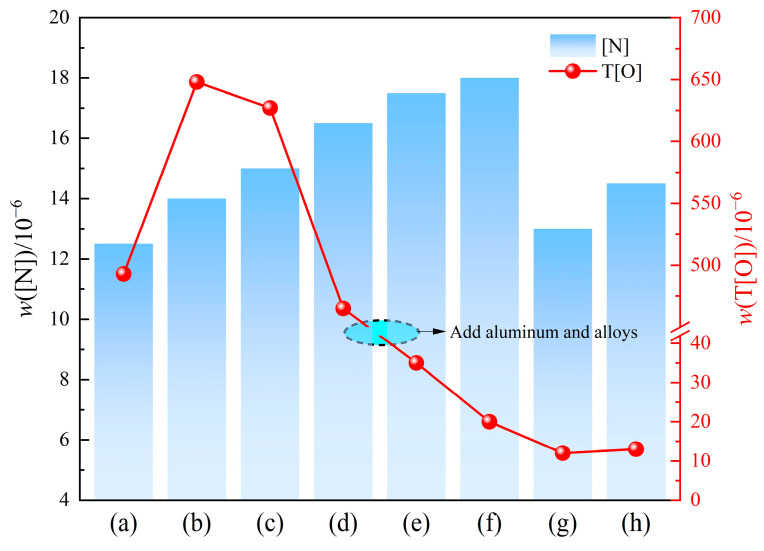
Variation in total oxygen and nitrogen mass fraction in molten steel during smelting (T ≥ 2000 °C, relative standard deviation ≈ 1%): (**a**) the end-point of converter blowing; (**b**) argon blowing station; (**c**) RH inlet; (**d**) after RH decarburization; (**e**) after RH deoxygenation and alloying; (**f**) RH desulfurization; (**g**) RH outbound; (**h**) tundish.

Figure 3 shows the variation in the average mass fraction of T[O] and [N] across four batches of steel samples throughout the entire smelting process of W350 non-oriented silicon steel. Overall, the mass fraction of T[O] generally presents a trend of increasing at first and then rapidly decreasing from the end-point of converter smelting to RH refining and subsequently to the tundish molten steel. The T[O] content in the molten steel before RH refining rises due to oxygen re-blowing and reactions with atmospheric oxygen. However, it declines at the beginning of RH vacuum refining, dropping sharply to approximately 0.0035% following RH deoxygenation and alloying (see Figure 3d). By the time the average mass fraction of T[O] in the RH outbound steel was a mere 0.0012%, it can be seen that the effectiveness of inclusion removal following aluminum deoxidation and alloying is evident. Furthermore, the vacuum circulation implemented after desulfurization enhances the floating and removal of inclusions. There is a slight increase in the molten steel *w*(T[O]) from the RH outbound to the tundish, which indicates that there is a secondary oxidation phenomenon in the molten steel throughout transportation or casting processes [20].

The average *w*([N]) in the molten steel at the end-point of converter blowing is approximately 0.00125%. As shown in Figure 3b, the *w*([N]) in the molten steel increases continuously during the alloy fine-tuning and refining processes, followed by a rise of 0.00055% after RH desulfurization. In addition, the *w*([N]) in the molten steel reaches 0.0013% after the RH vacuum net circulation, vacuum end, and RH outbound stages. Notably, the *w*([N]) in the molten steel increases by approximately 0.0001% to 0.0003% from the RH outbound to the tundish due to secondary oxidation phenomena. In order to prevent the nitrogen increase of the liquid steel in the subsequent continuous casting process, the sealing effect of the intrusive nozzle should be strengthened, and the entire pouring process should be protected.

### 3.2. Changes in Typical Inclusion Characteristics

#### 3.2.1. Evolution of Morphology and Chemical Composition of Typical Inclusions

**Figure 4:**
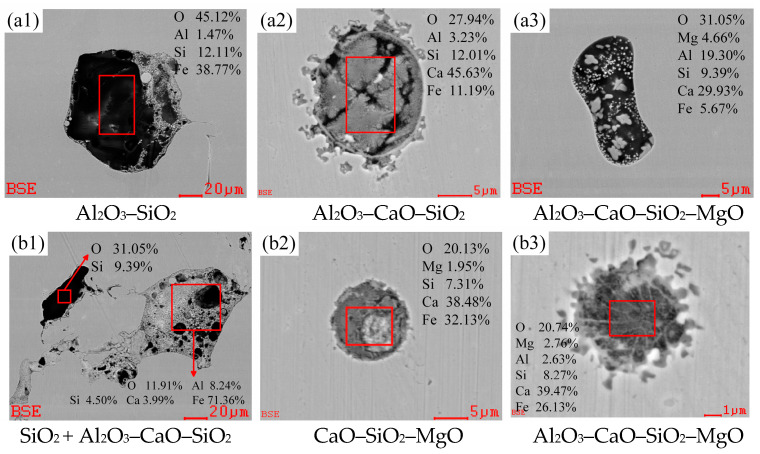
Typical inclusions before RH refining (at room temperature): (**a1**–**a3**) the end-point of converter blowing; (**b1**–**b3**) argon blowing station.

Figure 4 presents the SEM images of typical inclusions at the end-point of converter blowing and the argon blowing station for W350 non-oriented silicon steel. The SEM analysis revealed that the types of inclusions present in the molten steel before RH refining were relatively complex, and their sizes were notably large. At this stage, the main inclusions were cluster Al_2_O_3_–CaO–SiO_2_ inclusions (Figure 4(a1,b1)) and ellipsoidal Al_2_O_3_–CaO–SiO_2_–MgO inclusions (Figure 4(a3,b3)), in which the mass fraction of MgO and Al_2_O_3_ were relatively low and the diameter sizes were mostly between 5 and 20 μm. In addition, a few polygonal SiO_2_ inclusions with a length of about 30 μm and spherical CaO–SiO_2_–MgO inclusions with a diameter of about 5~10 μm were found in the argon blowing station (Figure 4(b1,b2)). It is preliminarily inferred that the actual oxygen content in the molten steel before RH refining is higher than the equilibrium oxygen content [21], resulting in a significant quantity and a complex variety of inclusions. Therefore, RH refining is necessary to further eliminate these inclusions, thereby enhancing the cleanliness of the molten steel. 

**Figure 5:**
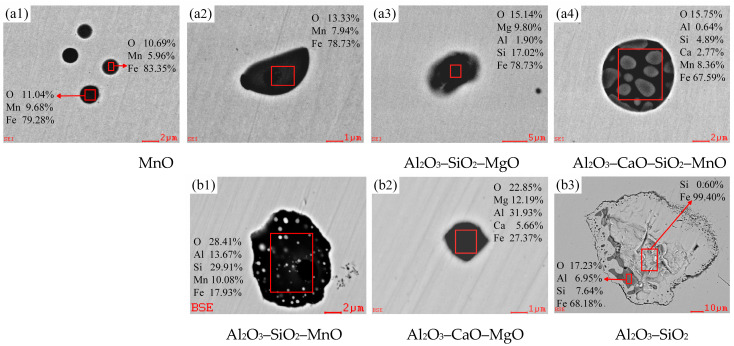

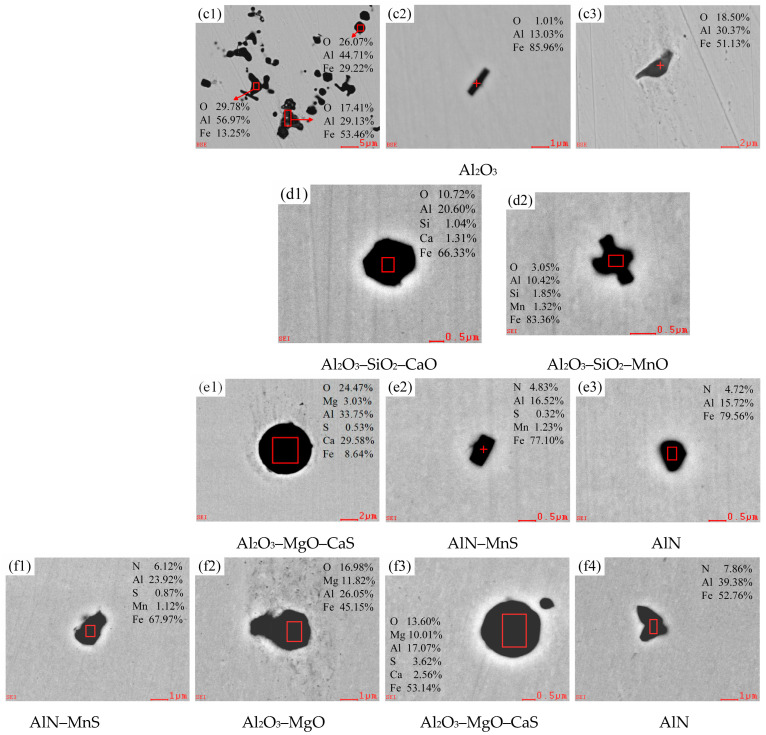
Typical inclusions during the RH refining process (at room temperature): (**a1**–**a4**) RH inlet; (**b1**–**b3**) RH decarburization for 3 min; (**c1**–**c3**) RH adding aluminum deoxygenation for 3 min; (**d1**,**d2**) RH for the first batch of alloying for 4 min; (**e1**–**e3**) RH desulfurization for 3 min; (**f1**–**f4**) RH outbound.

Figure 5 shows the morphological and chemical composition changes of typical inclusions at various stages of the RH refining process, as obtained through SEM and EDS analysis. It was observed that there is a large number of spherical MnO inclusions with diameters ranging from approximately 1 to 3 μm, as well as ellipsoidal Al_2_O_3_–SiO_2_–MgO and Al_2_O_3_–SiO_2_–MnO inclusions, which have a major axis measuring about 5 μm. In addition, there is a small number of near-spherical Al_2_O_3_–CaO–SiO_2_–MnO and Al_2_O_3_–CaO–MgO inclusions with diameters ranging from about 2 to 5 μm in the molten steel after RH inlet (Figure 5(a1–a4)) and decarburization for 3 min (Figure 5(b1–b3)). This is due to the low [Al] and high [Mn] in the molten steel of converter outbound, so that a large amount of [Mn] in the molten steel is oxidized, resulting in the formation of MnO and its composite inclusions during the decarburization process. After the addition of aluminum for 3 min (Figure 5(c1–c3)), due to the binding capacity of the Al element and O element being stronger than that of the Si element, the inclusions predominantly evolve into Al_2_O_3_, which is in the shape of clusters, spherical shapes, and elongated strips, with diameters and lengths primarily ranging from 2 to 5 μm.

In addition, after the addition of the first batch of ferrosilicon and electrolytic manganese metal for alloying (Figure 5(d1,d2)), the inclusions transform into polygonal Al_2_O_3_–SiO_2_–CaO and Al_2_O_3_–SiO_2_–MnO with sizes of the lengths ranging from approximately 1 to 3 μm, Notably, the mass fractions of SiO_2_ and MnO are observed to be lower in these inclusions. The addition of ferrosilicon and the significant reduction of MnO following the addition of aluminum are the primary factors influencing the transformation of inclusions. After the addition of the desulfurizer for 3 min, the inclusions were transformed into composite oxysulfides formed by various oxides and CaS [22], mainly spherical Al_2_O_3_–MgO–CaS inclusions with a diameter of about 5 μm (Figure 5(e1)). Additionally, a minor presence of both polygonal AlN–MnS and AlN inclusions, measuring around 1 μm in length, began to emerge (Figure 5(e2,e3)). AlN and MnS can not only combine with each other to form AlN–MnS composite inclusions, but they can also precipitate other composite inclusions through the nucleation of micron-sized oxides [23]. The RH outbound inclusions (Figure 5(f1–f4)) are mainly AlN with slightly larger size and some irregular polygonal Mg–Al spinels, which measure approximately 3 to 5 μm in length. The quantity of inclusions is significantly reduced after the molten steel undergoes thorough stirring via the vacuum net cycle, demonstrating a highly effective removal of inclusions.

**Figure 6:**
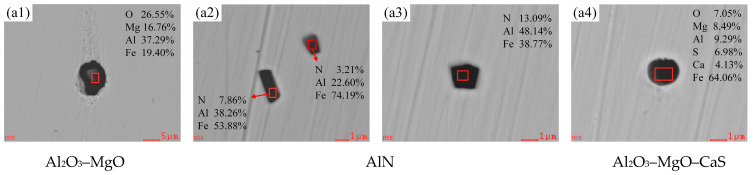
Typical inclusions in the tundish (at room temperature).

The generation of Al_2_O_3_–MgO significantly impacts the performance of finished steel. However, it can be determined that the raw materials added during the RH refining process do not contain magnesium. Many scholars [24,25] have noted that the Mg present in the non-metallic inclusions during the smelting process may originate from the MgO-containing dolomite used before the oxidation of carbon, phosphorus, aluminum, and silicon. It is more widely believed that it arises from the interfacial reactions between steel and slag, as well as the erosion of refractory materials by molten steel. Figure 6(a1–a4) shows the typical inclusions found in the tundish molten steel, which are mainly near-spherical Al_2_O_3_–MgO and polygonal AlN with arris or angles, along with a few elliptical Al_2_O_3_–MgO–CaS inclusions. The types and sizes of various inclusions in the tundish are basically the same as those in RH outbound, most of them are composed of deoxidation products in the molten steel and the lining or protective slag of the tundish. However, the number of inclusions has increased, which is consistent with the previous inference that there is a secondary oxidation phenomenon occurring in the tundish molten steel.

#### 3.2.2. Changes of Composition and Type in Oxide Inclusions

**Figure 7:**
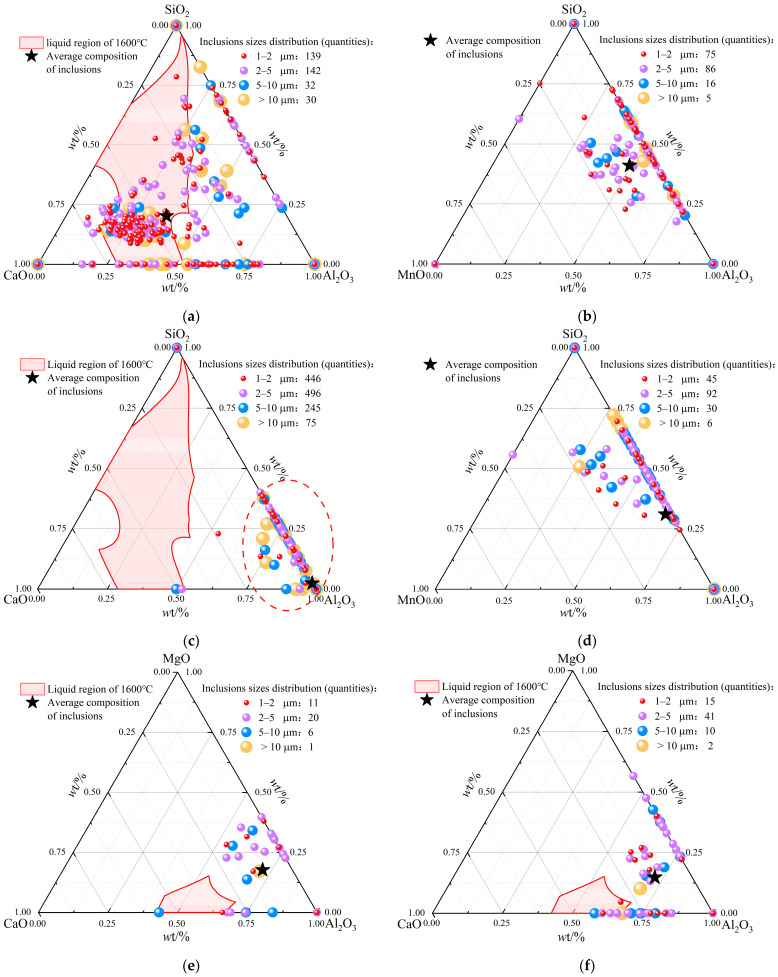
Distribution of oxide inclusions in different production processes: (**a**) before RH refining; (**b**) after RH decarbonization; (**c**) after RH deoxygenation; (**d**) after RH alloying; (**e**) RH outbound; (**f**) tundish.

In the smelting process of W350 non-oriented silicon steel, factors such as the addition of raw materials, the steel–slag reaction, the erosion of refractory materials, and secondary oxidation during transportation can significantly influence the composition of oxide inclusions. Therefore, it is essential to thoroughly understand the compositional changes of oxide inclusions throughout the smelting process to effectively control the cleanliness of molten steel. To quantitatively analyze these compositional changes, the content of each element, as determined by ASPEX, is converted into the corresponding compound content based on the conservation of metal elements [14]. This approach is widely employed in the quantitative study of inclusion compositions [11,26]. In the experiment, inclusions screened from each sample under diverse smelting processes were carefully projected into the ternary phase diagrams of Al_2_O_3_–CaO–SiO_2_, Al_2_O_3_–SiO_2_–MnO, and Al_2_O_3_–CaO–MgO. The liquidus at 1600 °C was drawn by Factsage calculation, in which the area surrounded by the liquidus was regarded as the low melting point area. Finally, the composition, quantity, and size of oxide inclusions in each sample were subjected to statistical classification, as shown in Figure 7.

According to Figure 7a, the oxide inclusion composition in the steel is randomly distributed in the low-melting-point region of the phase diagram before the RH refining. Based on the preceding analysis, these inclusions can be determined as clusters of Al_2_O_3_–CaO–SiO_2_ (Figure 4(a2,b1)) and Al_2_O_3_–CaO, with average mass fractions of Al_2_O_3_, CaO, and SiO_2_ in the phase diagram being 36%, 44%, and 20%, respectively. The [Mn] in the molten steel is oxidized to MnO in a large amount from the RH inlet to the RH decarburization process, which increases the mass fraction of MnO in the inclusions. The oxide inclusions formed after RH decarburization (Figure 7b) are mainly transformed into Al_2_O_3_–SiO_2_–MnO (Figure 5(b1)). At this stage, the average mass fraction proportion of Al_2_O_3_, SiO_2_, and MnO is approximately 5:4:1, respectively. After aluminum deoxidation, the inclusions in the steel are concentrated at the rightmost part of the Al_2_O_3_–CaO–SiO_2_ phase diagram (Figure 7c). At this stage, the concentration of MnO is significantly reduced. The inclusions primarily consist of Al_2_O_3_ and exhibit various shapes (Figure 5(c1–c3)) during the processes of aggregation and floating.

With the RH refining, the mass fractions of [Si] and [Mn] in the molten steel increase following the addition of ferrosilicon and manganese metal (Figure 7d). The inclusions within the steel primarily consist of Al_2_O_3_–SiO_2_–MnO (Figure 5(d2)), along with some Al_2_O_3_ inclusions that were not completely eliminated. Additionally, during the RH desulfurization process, CaO in desulfurizers, such as lime powder, is reduced to Ca, which then reacts with S in the molten steel to produce a significant amount of CaS that adheres to the surface of Al_2_O_3_–MgO [15], resulting in the formation of Al_2_O_3_–MgO–CaS inclusions (Figure 5(e1)). After the addition of the desulfurizer, the molten steel is stirred by vacuum net circulation, so that the inclusions are fully floated into the slag for removal. The number of inclusions is extremely reduced in RH outbound. At this stage, the inclusions primarily consist of Al_2_O_3_–MgO and Al_2_O_3_–MgO–CaS (Figure 5(f1–f4)), as well as a portion of the inclusions being composed of calcium aluminate (Figure 7e). Figure 7f indicates that the mass fraction of MgO within the inclusions of the molten steel in the tundish was decreased. This shift results in an overall movement of the phase diagram composition towards the region of lower melting points. In the course of this production process, the types of inclusions remain unchanged, and the number of inclusions slightly increases compared to RH outbound. It can be inferred that the inadequate sealing of the upper nozzle in the tundish, along with the entrapment of slag in the molten steel, leads to a deterioration in cleanliness.

Combined with the above analysis, Table 3 shows various typical inclusions in the smelting process of W350 non-oriented silicon steel. It mainly includes large-sized Al_2_O_3_–CaO–SiO_2_ and Al_2_O_3_–CaO–SiO_2_–MgO generated by converter smelting and slag entrapment in the argon station, MnO and Al_2_O_3_–SiO_2_–MnO generated by RH decarburization process, Al_2_O_3_ generated by adding aluminum deoxidization, transformed into Al_2_O_3_–SiO_2_–MnO after alloying, and Al_2_O_3_–CaO–SiO_2_ and Al_2_O_3_–MgO–CaS generated by RH desulfurization, as well as Al_2_O_3_–MgO–CaS, Al_2_O_3_–MgO, AlN, and other inclusions detected in the RH outbound and the tundish. There are still very few single-phase Al_2_O_3_ inclusions present in the tundish. The analysis indicates that the overall number of inclusions in molten steel has significantly decreased from converter smelting to RH refining and subsequently to the tundish; however, the types of inclusions have gradually increased. Therefore, controlling the cleanliness of molten steel typically involves multiple processes, including converter blowing, RH vacuum refining, top slag composition control, tundish metallurgy, and protective casting. Any errors in any specific link of this entire process may lead to an increase in inclusions, thereby affecting the performance of the finished product.

#### 3.2.3. Changes of Size and Quantity in Oxide Inclusions

**Figure 8:**
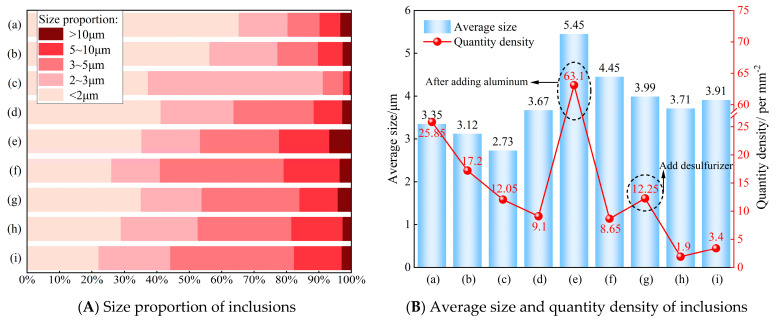
Variation in size and quantity of inclusions in molten steel under different production processes: (**a**) the end-point of converter blowing; (**b**) argon blowing station; (**c**) RH inlet; (**d**) after RH decarbonization; (**e**) after RH deoxygenation; (**f**) after RH alloying; (**g**) after RH desulfurization; (**h**) RH outbound; (**i**) tundish.

ASPEX can be used to automatically detect all inclusion information that meets the set conditions on a plane after grinding and polishing of steel samples, including size (≥1 μm), composition, area, and position [14]. Figure 8 illustrates the changes in the size and quantity of oxide inclusions in steel samples taken from W350 non-oriented silicon steel throughout the entire smelting process. As shown in Figure 8A, approximately 80% of the inclusions during the entire process size are smaller than 5 μm; notably, over 90% of the inclusions before RH refining are less than 3 μm. With the RH refining process, the size of inclusions in the steel increased following collision and polymerization, and then both the average size and the number per unit area of inclusions reached the maximum values after RH deoxidation, measuring 5.45 μm and 63.1 per mm^2^, respectively (Figure 8B). This is due to the fact that a great number of Al_2_O_3_ inclusions in steel do not float adequately during the collision and aggregation process following the addition of aluminum pellets for deoxidation. Furthermore, the insufficient circulation of molten steel after the introduction of deoxidizers and desulfurizers has resulted in varying degrees of rebound in the number density of inclusions. Overall, the number density of inclusions has exhibited a downward trend. The average size and density of inclusions in RH outbound are 3.71 μm and 1.9 per mm^2^, respectively, after sufficient circulation of the molten steel. In the tundish, the average size and number density of inclusions show a slight increase, with very few inclusions exceeding 10 μm in size. This observation is consistent with the earlier conclusion that a secondary oxidation phenomenon occurs in molten steel during transportation or pouring.

### 3.3. Analysis of Formation Mechanism of Typical Inclusions

High-grade non-oriented silicon steel has stringent requirements regarding iron loss and magnetic induction strength. In particular, the control of steel cleanliness [27] and inclusion level [17] is a critical factor in the development of high-grade non-oriented silicon steel varieties. The Al_2_O_3_, Al_2_O_3_–MgO, and calcium aluminate inclusions present during the smelting process of W350 non-oriented silicon steel exhibit characteristics such as high melting points, high hardness, and resistance to deformation during the rolling process. Therefore, it is relatively easy for deposits to form on the inner wall of the nozzle, potentially leading to blockage and impeding the smooth flow of production. In addition, research shows that the residual inclusions can easily cause surface defects in the rolling process [16,28]. Precipitates such as AlN and MnS not only indirectly inhibit grain growth but also induce lattice distortion, leading to defects such as dislocations and vacancies. Additionally, they hinder the alteration of the magnetic domain structure. The increased magnetization resistance results in elevated hysteresis loss, which significantly impacts the magnetic properties of the finished silicon steel [29,30]. Therefore, it is crucial to investigate the formation and transformation mechanisms of typical inclusions in non-oriented silicon steel to effectively control the morphology of inclusions and enhance the magnetic properties of the finished electrical steel. In the thermodynamic calculation of the experiment, because part of the thermodynamic data and calculation formula are simplified, when the simplified model is established for calculation, simplifying assumptions might influence the results. This may lead to a bias in the calculation results. Addressing these limitations would significantly enhance the transparency and reliability of the conclusions.

#### 3.3.1. Thermodynamic Analysis of Al_2_O_3_–MgO Formation

The formation of spinel occurs as a result of the interactions among molten steel, slag (furnace lining), and inclusions. This paper analyzes the conditions for spinel formation during the smelting process of W350 non-oriented silicon steel by integrating the stable phase diagram of the MgO/Al_2_O_3_–MgO/Al_2_O_3_ system, which was derived from thermodynamic calculations. The relationship between activity and the activity coefficient utilized in these calculations is shown in Equation (1). Additionally, Equation (2) describes the relationship between the activity coefficient, the interaction coefficient, and the mass fraction of the elements, as determined by the Wagner model. The relevant reaction equations pertaining to the Mg–Al–O system are shown in Table 4 [31].

(1) $$a_{i}=f_{i}\cdot w\left(\left[i\right]\right)$$

(2) $$\mathrm{lg}f_{i}=\sum_{j}^{n}e_{i}^{j}w\left(\left[j\right]\right)$$

where *a_i_* and *f_i_* refer to the activity and activity coefficient of component i, respectively, and *i* and *j* represent components in molten steel. *w*([*i*]) and *w*([*j*]) refer to the mass fraction of components *i* and *j*, respectively. $e_{i}^{j}$ refers to the first-order interaction coefficient between components in molten steel.

(1) The boundary of the MgO/Al_2_O_3_–MgO phase is as follows:

(3) $$4(\mathrm{MgO})+2[\mathrm{Al}]=\mathrm{MgO}\cdot {\mathrm{Al}}_{2}O_{3}\left(s)+3[\mathrm{Mg}\right]$$

(4) $$\mathrm{lg}K_{1}=-33.09+50,880/T$$

(5) $$K_{1}=\frac{a_{\mathrm{MgO}\cdot {\mathrm{Al}}_{2}O_{3}}\cdot \;a_{\left[\mathrm{Mg}\right]}^{3}}{a_{\mathrm{MgO}}^{4}\cdot a_{\left[\mathrm{Al}\right]}^{2}}=\frac{a_{\mathrm{MgO}\cdot {\mathrm{Al}}_{2}O_{3}}\cdot f_{\mathrm{Mg}}^{3}\cdot \;{(w\left([\mathrm{Mg}])\right)}^{3}}{a_{\mathrm{MgO}}^{4}\cdot f_{\mathrm{Al}}^{2}\;\cdot \;{(w\left([\mathrm{Al}])\right)}^{2}}$$

(2) The boundary of the Al_2_O_3_–MgO/Al_2_O_3_ phase is as follows:

(6) $$3\mathrm{MgO}\cdot {\mathrm{Al}}_{2}O_{3}(s)+2[\mathrm{Al}]=4({\mathrm{Al}}_{2}O_{3})+3[\mathrm{Mg}]$$

(7) $$\mathrm{lg}K_{2}=-34.37+46,950/T$$

(8) $$K_{2}=\frac{a_{{\mathrm{Al}}_{2}O_{3}}^{4}\cdot \;a_{\left[\mathrm{Mg}\right]}^{3}}{a_{\mathrm{MgO}\cdot {\mathrm{Al}}_{2}O_{3}}^{3}\cdot a_{\left[\mathrm{Al}\right]}^{2}}=\frac{a_{{\mathrm{Al}}_{2}O_{3}}^{4}\cdot f_{\mathrm{Mg}}^{3}\cdot \;{(w\left([\mathrm{Mg}])\right)}^{3}}{a_{\mathrm{MgO}\cdot {\mathrm{Al}}_{2}O_{3}}^{3}\cdot f_{\mathrm{Al}}^{2}\;\cdot \;{(w\left([\mathrm{Al}])\right)}^{2}}$$

where K and *T* refer to the chemical equilibrium constant and reaction temperature, respectively.

**Figure 9:**
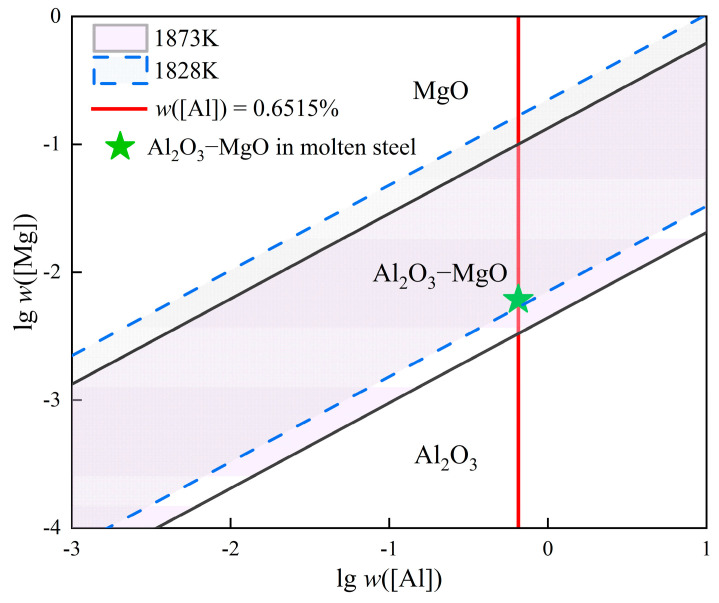
Phase equilibrium diagram of MgO/Al_2_O_3_–MgO/Al_2_O_3_ at RH outbound.

Table 5 shows the first-order interaction coefficients among the components in the molten steel [32]. According to the literature [33], when the MgO/Al_2_O_3_–MgO phase boundary is calculated at 1873 K, the activities of Al_2_O_3_–MgO and MgO are taken as 0.8 and 0.99, respectively. For the calculation of the Al_2_O_3_–MgO/Al_2_O_3_ phase boundary, the activities of Al_2_O_3_–MgO and Al_2_O_3_ are considered to be 0.47 and 1, respectively. In this experiment, the composition of the molten steel in the RH outbound, as detailed in Table 2, was selected for the aforementioned calculations. The results of these calculations are illustrated in Figure 9.

According to Figure 9, when the slag and molten steel are in equilibrium at a temperature of 1873 K and the *w*([Al]s) in steel is 0.0001%, as long as *w*([Mg]) > 9.503 × 10^−6^, the Al_2_O_3_ inclusions in steel will gradually transform into Al_2_O_3_–MgO inclusions. Therefore, as long as there is a tiny amount of magnesium and aluminum in steel, Al_2_O_3_–MgO can be generated at each stage of the smelting process in theory. With the increase in the aluminum mass fraction in molten steel, the magnesium mass fraction required for the formation of Al_2_O_3_–MgO also gradually increases [15]. The mass fraction of dissolved aluminum in W350 non-oriented silicon steel produced in the factory is typically controlled at 0.64 to 0.70% during RH outbound processing. Consequently, Figure 9 indicates that the required *w*([Mg]) for the formation of Al_2_O_3_–MgO ranges from 0.0032% to 0.0056%.

The actual *w*([Al]s) in the RH outbound molten steel measured by ICP-AES is 0.6515% (Table 2), which is projected in Figure 9. It can be observed that Al_2_O_3_–MgO inclusions are generated when the temperature reaches 1873 K and lg*w*([Mg]) exceeds −2.4795, that is, *w*([Mg]) exceeds 0.0033%. Assuming that the interaction coefficient and activities of each component in the molten steel remain relatively constant, the range of *w*([Mg]) corresponding to the stable existence of Al_2_O_3_–MgO is between 0.0053% and 0.1676% at the actual temperature of 1828 K during this industrial test. The actual Al_2_O_3_–MgO inclusion in this test steel is shown as the green star shape in Figure 9. It is evident that as the temperature of the molten steel decreases, the magnesium mass fraction required to generate Al_2_O_3_–MgO gradually increases, although the increase is relatively small. It is inferred that the addition of ferrosilicon alloy during the alloying process, along with the circulation of molten steel, facilitates the further reduction of magnesium from the slag and refractory materials into the molten steel, leading to the formation Al_2_O_3_–MgO inclusions. As mentioned above, Al_2_O_3_–MgO inclusions bound by CaS (Figure 5(e1)) have been identified following RH alloying and desulfurization, while Al_2_O_3_–MgO inclusions (Figure 5(f2) and Figure 6(a1)) are commonly observed in RH outbound and tundish, which aligns with the earlier results from experimental tests.

#### 3.3.2. Thermodynamic Analysis of the Second-Phase Precipitation

**Figure 10:**
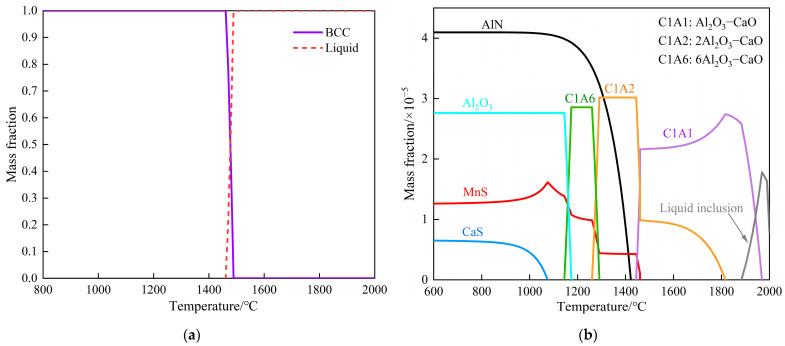
Transformation of precipitates during solidification process: (**a**) phase transition; (**b**) compositional transformation.

The liquidus temperature of W350 non-oriented silicon steel, along with the precipitation temperature of typical inclusions, was calculated using Thermal-Calc software and its supporting TCFE13 database [34]. This was accomplished by inputting the chemical composition of the tundish molten steel as presented in Table 2. The results are shown in Figure 10.

Figure 10 indicates that the liquidus temperature of W350 non-oriented silicon steel calculated by Thermal-Calc is 1489 °C, and the matrix is ferrite α phase after solidification. At temperatures exceeding 1600 °C, inclusions predominantly exist in a liquid phase, with calcium aluminate inclusions mainly manifesting as C1A1 (Al_2_O_3_–CaO) and C1A2 (2Al_2_O_3_–CaO) types. As the temperature decreases to 1460.7 °C, the MnS phase initiates precipitation. When the temperature further reduces to 1444.3 °C, the precipitation of Al_2_O_3_–CaO inclusions ceases, and most of these inclusions transition into 2Al_2_O_3_–CaO inclusions. At a temperature of 1422.2 °C, the AlN phase begins to precipitate. As the temperature continues to decrease to 1290.9 °C, C1A6 (6Al_2_O_3_–CaO) inclusions start to form. Subsequently, at 1259.8 °C, the precipitation of 2Al_2_O_3_–CaO inclusions halts, and these inclusions predominantly convert into 6Al_2_O_3_–CaO inclusions. Throughout the temperature range of 1000°C to 2000 °C, the sequence of inclusion transformation is as follows: liquid inclusion → Al_2_O_3_–CaO → 2Al_2_O_3_–CaO + MnS → 6Al_2_O_3_–CaO → Al_2_O_3_ + AlN + MnS + CaS.

In this experimental study, the presence of AlN–MnS and AlN inclusions was initially detected following the desulfurization process in molten steel. It was considered that the AlN and MnS inclusions were formed by local solidification or cooling with the addition of the desulfurizer during the RH desulfurization process. The high-temperature conditions of the RH refining process accelerate the diffusion rate of nitrogen and aluminum, leading to the precipitation of numerous AlN inclusions. These inclusions typically form composite structures with Al_2_O_3_ as the core after aggregation and growth [35]. However, no single-phase MnS inclusions were detected throughout the smelting process. It was posited that the kinetic conditions during smelting were insufficient to facilitate the homogeneous nucleation of MnS; thus, MnS could only nucleate and grow on the surface of other inclusions through heterogeneous nucleation [15,36], which aligns with the previously observed and detected results.

In summary, it can be concluded that the magnetic properties of non-oriented silicon steel are intricately linked to the presence of second-phase particles within the steel. The fine inclusions and precipitates present in the steel exert a significant influence on the iron loss and magnetic induction properties of the finished silicon steel [37]. Consequently, one of the critical research directions is to enhance the cleanliness of non-oriented silicon steel by precisely controlling the types and quantities of inclusions and precipitates present in the steel, thereby mitigating their impact on grain growth and the motion of magnetic domain walls.

## 4. Conclusions

(1) This factory employs aluminum deoxidation during the RH refining process of W350 non-oriented silicon steel. The average mass fractions of total oxygen and nitrogen decrease significantly during the RH refining stage, reaching their lowest values in the RH outbound, which are 0.0012% and 0.0013%, respectively. However, a secondary oxidation phenomenon occurs during the tundish smelting stage, resulting in a final nitrogen mass fraction that is slightly above the target value. This indicates a need for further enhanced protection during the pouring process.

(2) During the smelting process of W350 non-oriented silicon steel, the end-points of the converter and the argon blowing station primarily consist of large-sized cluster Al_2_O_3_–CaO–SiO_2_ and Al_2_O_3_–CaO–SiO_2_–MgO composite inclusions. The inclusions after the RH inlet are mainly small-sized spherical MnO inclusions. After RH decarburization, the mass fraction of MnO in the inclusions increases, leading to the dominance of ellipsoidal Al_2_O_3_–SiO_2_–MnO inclusions. Subsequent RH deoxidation with aluminum results in the inclusions in the steel concentrating on the Al_2_O_3_ side of the Al_2_O_3_–CaO–SiO_2_ phase diagram. During this process, the inclusions are largely transformed into irregular Al_2_O_3_ inclusions ranging from 2 to 5 μm. After RH alloying, the mass fractions of [Si] and [Mn] in molten steel increase, resulting in the transformation of inclusions into polygonal Al_2_O_3_–SiO_2_–MnO inclusions. Additionally, spherical Al_2_O_3_–MgO–CaS, polygonal AlN with arris or angles, and near-spherical Al_2_O_3_–MgO inclusions were observed following RH desulfurization, in RH outbound, or in the tundish. Notably, the size of Al_2_O_3_–MgO inclusions is relatively large, and the content of MgO in these inclusions tends to increase throughout the smelting process.

(3) The number of inclusions during the smelting process decreases continuously. Approximately 80% of the oxide inclusions throughout the entire process measure less than 5 μm in size. After RH deoxidation, the average size and the number per unit area of inclusions in the steel reached their maximum values of 5.45 μm and 63.1 per mm^2^, respectively, which can be attributed to the insufficient floating of inclusions. Furthermore, the overall number density of inclusions exhibited a downward trend. The average size and number per unit area of inclusions decreased to their minimum values during the RH outbound phase, measuring 3.71 μm and 1.9 per mm^2^, respectively. Meanwhile, the average size and number per unit area of inclusions in the tundish slightly increased. Consequently, it is essential to enhance the sealing effect of the upper nozzle of the tundish to prevent slag entrapment and avoid secondary oxidation of the molten steel.

(4) The results of thermodynamic calculation indicate that Al_2_O_3_–MgO inclusion is easily generated during the smelting process in theory. The *w*([Al]s) in the RH outbound steel ranges from 0.64% to 0.70%, which corresponds to the *w*([Mg]) required for the formation of Al_2_O_3_–MgO between 0.0032% and 0.0056% at 1873 K. In this industrial test, with an actual temperature of 1828 K and *w*([Al]s) at 0.6515%, the stable range of *w*([Mg]) corresponding to the existence of Al_2_O_3_–MgO is found to be between 0.0053% and 0.1676%. It is calculated that the liquidus temperature of W350 non-oriented silicon steel is 1489 °C. As the temperature decreases, the precipitation of MnS and AlN inclusions occurs successively, with precipitation temperatures of 1460.7 °C and 1422.2 °C, respectively. The calculated sequence of inclusion precipitation is as follows: liquid inclusion → Al_2_O_3_–CaO → 2Al_2_O_3_–CaO + MnS → 6Al_2_O_3_–CaO → Al_2_O_3_ + AlN + MnS + CaS.

(5) Through the analysis and discussion of the full text, it is evident that W350 non-oriented silicon steel can achieve better inclusion control following RH refining, and the cleanliness of molten steel is high. However, there is a secondary oxidation phenomenon during actual production. To address this issue, immersion nozzles, ladle capping, and the addition of appropriate covering agents can be employed to protect the casting. Additionally, the tundish is equipped with a retaining wall, and electromagnetic stirring is utilized to regulate the flow of molten steel and the movement of inclusions, thereby enhancing the cleanliness of the molten steel.

## Figures and Tables

**Table 1:** Main chemical composition of W350 non-oriented silicon steel in tundish (wt%).

C	Si	Mn	P	S	Als	Ti	N
≤0.0020	3.30~3.40	0.45~0.55	≤0.020	≤0.0010	0.60~0.70	≤0.0015	≤0.0010

**Table 2:** Changes in molten steel composition during smelting (wt%).

Process Nodes	C	Si	Mn	P	S	Als	Ca	N	O
RH outbound	0.0018	3.3138	0.4940	0.0137	0.0010	0.6515	0.0010	0.0012	0.0012
Tundish	0.0019	3.3181	0.4992	0.0138	0.0008	0.6481	0.0007	0.0014	0.0013

**Table 3:** Types of typical inclusions during the whole smelting.

Process Nodes	Main Inclusions	Partial Inclusions
The end-point of converter blowing	Al_2_O_3_–CaO–SiO_2_, Al_2_O_3_–CaO–SiO_2_–MgO	Al_2_O_3_–CaO
Argon blowing station	Al_2_O_3_–CaO–SiO_2_	SiO_2_, CaO–SiO_2_–MgO
RH inlet	MnO, Al_2_O_3_–SiO_2_–MgO	Al_2_O_3_–CaO–SiO_2_–MnO
After RH decarbonization	Al_2_O_3_–SiO_2_–MnO	Al_2_O_3_–CaO–MgO
After RH deoxygenation	Al_2_O_3_	Al_2_O_3_–CaO–SiO_2_
After RH alloying	Al_2_O_3_–SiO_2_–MnO	Al_2_O_3_–CaO–SiO_2_
After RH desulfurization	Al_2_O_3_–MgO–CaS	AlN–MnS, AlN
RH outbound	AlN, Al_2_O_3_–MgO	Al_2_O_3_–MgO–CaS, AlN–MnS
Tundish	Al_2_O_3_–MgO, AlN	Al_2_O_3_–MgO–CaS

**Table 4:** Gibbs Free Energy of main reactions [31].

Reaction Equation	Log K
2[Al] + 3[O] = Al_2_O_3_	−20.57 + 64,000/*T*
[Mg] + [O] = MgO	4.28 + 4700/*T*
MgO + Al_2_O_3_ = MgO•Al_2_O_3_	0.32 + 980/*T*

**Table 5:** First-order interaction coefficients of components in molten steel at 1873K [32].

$e_{i}^{j}$	C	Als	Si	Mn	P	S	Ca	N	O
Al	0.091	0.045	0.0056	−0.004	0.033	0.030	−0.047	−0.058	−6.60
Mg	−0.240	−0.270	−0.096	—	—	−1.380	—	—	−460

## Data Availability

Data are contained within the article.
